# The Molecular Mechanism Investigation of HBP‐A Slows Down Meniscus Hypertrophy and Mineralisation by the Damage Mechanical Model

**DOI:** 10.1111/jcmm.70271

**Published:** 2024-12-10

**Authors:** Zongrui Yang, Yuanyuan Feng, Mingcai Zhang, Yongming Liu, Yizhe Xiong, Xiang Wang, Ying Shi, Bo Chen, Zhengming Wang, Haiya Ge, Hongsheng Zhan, Zhibi Shen, Guoqing Du

**Affiliations:** ^1^ Shi's Center of Orthopedics and Traumatology Shuguang Hospital Affiliated to Shanghai University of Traditional Chinese Medicine Shanghai China; ^2^ Institute of Traumatology & Orthopedics Shanghai Academy of Traditional Chinese Medicine Shanghai China; ^3^ Department of Medical Oncology Shuguang Hospital Affiliated to Shanghai University of Traditional Chinese Medicine Shanghai China

**Keywords:** abnormal mechanical damage, HBP‐A, hypertrophy and mineralisation, KOA, meniscus, p38‐MAPK signalling pathway

## Abstract

HBP‐A is the main active component of a traditional Chinese medicine *Huaizhen Yanggan Capsule,* for the remarkable treatment of knee osteoarthritis (KOA). This study aimed to elucidate the ameliorative effect of HBP‐A on meniscus hypertrophy and mineralisation in KOA and the molecular mechanism of its action. An Hartley guinea pig model of KOA that underwent anterior cruciate ligament transection (ACLT) and a model of rat primary meniscus fibrochondrocytes (PMFs) were used to investigate the ameliorative effect of HBP‐A on meniscal hypertrophy and calcification and its signal transduction mechanism of action. The results show that Guinea pig's meniscus width, as well as the area of meniscus calcification and meniscus and articular cartilage injury score, were significantly reduced in the HBP‐A intervention group compared to the ACLT group. The expression levels of mtrix metalloproteinase 13 (MMP13), runt‐related transcription factor 2 (Runx2), Indian hedgehog (Ihh), alkaline phosphatase (ALP), and ankylosis homologue (ANKH) at the protein and gene level significantly decreased in the HBP‐A intervention group compared to the ACLT group. In vitro study, apoptosis, hypertrophy, and calcification of rat PMFs after 10% stretch force were significantly improved with HBP‐A intervention. Western blot and RT‐qPCR showed that hypertrophy, calcification, and p38 MAPK signalling pathway‐related markers of PMFs were incredibly depressed in the HBP‐A intervention group compared to the 10% stretch force group. In conclusion, HBP‐A can slow down meniscus hypertrophy and mineralisation induced by abnormal mechanical loading, and its mechanism of action may be through the p38‐MAPK signalling pathway.

## 1 Introduction

The knee has become a high‐incidence site for osteoarthritis due to its complex anatomy [1]. KOA is a clinically typical chronic bone and joint disease that imposes a substantial socio‐economic burden on individuals and the nation [2]. The pathogenesis and prevention of KOA have become a significant focus of research and a hot issue for medical practitioners in this field. Trauma, sports injury, obesity, and advanced age are recognised as important causes of KOA [3, 4, 5], all of which can lead to an expected outcome—knee mechanical imbalance. A mechanical imbalance alters the knee's stability, muscle morphology, and mobility, increasing abnormal mechanical loading of the tissues within the joint, which further aggravates KOA [6, 7, 8]. However, current research on the development and progression of KOA induced by abnormal mechanical damage has focused on the articular cartilage [5, 9, 10, 11]. The importance of other structures is often overlooked.

The meniscus, as one of the essential structures of the knee joint, plays an integral role in the development of KOA. The meniscus can mechanically transfer, absorb shock, and maintain joint stability [12, 13, 14, 15]. After the meniscal injury, the joint often undergoes biomechanical changes. The ability of the damaged meniscus to transfer loads in the joint is reduced, and the abnormal mechanical loads caused by joint instability can, in turn, exacerbate damage to other tissues and structures in the joint, leading to a degeneration of the knee joint and even post‐traumatic osteoarthritis (PTOA) [16, 17]. Therefore, it may play a ‘bridging’ role in developing and progressing abnormal mechanical loading of the joint and osteoarthritis. It has been demonstrated that abnormal mechanical damage leads to meniscal tissue cell death, loss of proteoglycans, and production of matrix‐degrading enzymes [17, 18], which suggests that abnormal mechanical damage can exacerbate meniscal degeneration. At the same time, degeneration of the meniscus can also damage the articular cartilage, which is an important factor in causing or aggravating KOA [19, 20, 21]. Related reports exhibited that the presence of calcification in the meniscus of patients with KOA, which causes narrowing of the joint space and friction with the cartilage, is a critical pathological factor in the development and progression of KOA [22, 23]. Our previous study on a guinea pig model of knee mechanical injury also confirmed that abnormal mechanical loading leads to excessive meniscal hypertrophy and calcification, which contributes to KOA's development [22]. This suggests that meniscal hypertrophy and calcification due to abnormal mechanical damage play an essential role in developing KOA. Inhibition of meniscus pathological degeneration has become a potential new therapeutic target for KOA.

Previous studies have shown that abnormal mechanical loading can lead to phosphorylation and upregulation of p38 protein expression in cells, which activates the p38‐MAPK signalling pathway [23, 24, 25]. However, whether the activation of the p38‐MAPK signalling pathway by abnormal mechanical loading can further change the expression of the markers associated with meniscal hypertrophy and calcification has yet to be reported. It has been reported that p38‐MAPK induces degradation of the HDAC4 by upregulating the expression of its downstream substrate caspase‐3, which in turn increases the expression of Runx2 and MMP13, markers associated with pathological hypertrophy and calcification of meniscus, respectively [26, 27, 28]. The activation of the p38‐MAPK signalling pathway by abnormal mechanical damage leads to the degradation of HDAC4 by upregulating the expression of its downstream substrate caspase‐3, which in turn upgrades its expression of MMP13 and Runx2, leading to excessive hypertrophy and calcification of the meniscus. This is the focus of our research.

Huaizhen Yanggan Capsule (Patent No. ZL200610027835.1) is a hospital preparation independently developed by Shuguang Hospital affiliated to Shanghai University of Traditional Chinese Medicine for the clinical treatment of KOA. The major component of that compound is HBP‐A from Anodonta (Patent No. ZL200610028598.0). HBP‐A is a kind of a‐glucan composing. The study documented that the potential pharmacological target of glucan HBP‐A in chondrocyte monolayer culture and tissue engineered cartilage in vivo may be concerned with the inhibition of catabolic enzymes MMP3, ADAMTs‐5, and increasing of type II collagen expression [29].

This study aimed to investigate the effects of abnormal mechanical damage on the p38‐MAPK signalling pathway as well as the main markers of pathological hypertrophy and calcification of meniscus, and the role of HBP‐A in the regulation of the p38‐MAPK signalling pathway and whether it can delay or inhibit excessive hypertrophy and calcification of the meniscus caused by abnormal mechanical damage. Based on the previous study, we propose the hypothesis that (1) abnormal mechanical loading activates the p38‐MAPK signalling pathway and leads to the degradation of HDAC4 by upregulating the expression of its downstream substrate caspase‐3, which is an essential molecular biological mechanism for the upregulation of markers associated with hypertrophy and calcification in the meniscus; (2) the active ingredient of mussel meat, HBP‐A, acts to slow down or inhibit excessive meniscal hypertrophy and calcification as well as cartilage protection by regulating the upstream and downstream targets of the p38‐MAPK signalling pathway. The study will be conducted both in vitro and in vivo.

## 2 Materials and Methods

### 2.1 Preparation of HBP‐A

HBP‐A was obtained from anodonta meat through a process involving boiling, centrifugation, extraction, freeze‐drying, and chromatography, as detailed in Patent No. ZL200610028598.0.

### 2.2 In Vivo Guinea Pig Study

#### 2.2.1 Animal Experimental Design

The study was approved by the National Natural Science Foundation of China (82174403). Approval for the animal experiments was obtained from the Institutional Animal Care and Use Committee at Shanghai University of Traditional Chinese Medicine (Ethical Approval No. PZSHUTCM220913022). Three‐month‐old male Hartley guinea pigs (*n* = 40) were purchased and maintained in Shanghai University of Chinese Medicine Laboratory Animal Centre under controlled conditions of temperature (22°C ± 2°C) in a light/dark cycle of 12 h:12 h.

Mechanical damage model of knee joint is procedure by surgery. Thirty‐two guinea pigs underwent ACLT [22] on the right knee, while the contralateral ACL‐intact (the left) knee served as a sham control. Three days after the model was made successful, 200 μL of different doses of HBP‐A were injected into the right knee joint cavity. The HBP‐A intervention group was divided into three different concentration groups: low concentration group (*n* = 8, 7.5 mg/mL), middle concentration group (*n* = 8, 15 mg/mL), and high concentration group (*n* = 8, 30 mg/mL). Thereafter, HBP‐A was injected twice a week for 10 weeks.

At the end of the treatment, the guinea pigs were euthanised by an overdose of carbon dioxide according to the American Veterinary Medical Association (AVMA) guidelines [30], then the menisci and tibia of the right (ACLT) and left (control) knees was individually harvested from the animals. The menisci and tibial cartilage are photographed, and the meniscal width from medial to lateral were measured with a ruler (*n* = 8). Subsequently, the meniscus and tibia of half of the animals in each group were snap frozen in liquid nitrogen and stored at −80°C for molecular assays; the other half had their meniscus and tibia fixed in formalin for stainings.

#### 2.2.2 Histology

The menisci and tibial cartilages in each group (*n* = 4) were then immersed in 10% formalin for 72 h at room temperature. The tibial articular cartilage was decalcified in a 20% ethylenediaminetetraacetic acid (EDTA) solution while the meniscus was processed without decalcification. The specimens were then dehydrated in gradient alcohol, clear in xylene, embedded in paraffin, and sectioned at 6um for subsequent staining. Alizarin red and Von Kossa staining were used to evaluate the mineralisation of the meniscus. The area and intensity of mineralisation were quantified using HIS‐Elements AR software (Nikon). Safranin O/Fast Green staining was used to evaluate the damage to the meniscus and tibial articular cartilage. The severity of the meniscus damage was graded roughly [31], and the severity of the cartilage damage was determined by the Osteoarthritis Research Society International (OARSI) histological score [32]. Photomicrographs were obtained using a Nikon Ri1 microscope (Nikon Corporation).

#### 2.2.3 Immunohistochemistry

The right meniscus tissue section was used to evaluate the expression of protein markers MMP13, Runx2, Ihh, ALP, and ANKH associated with hypertrophy and mineralisation by immunohistochemistry. Immunohistochemical staining was performed using the 3, 3′ diaminobenzi dine (DAB) streptavidin‐peroxidase (SP) DAB Histostain‐SP immunohistochemistry kit (PA110; Tiangen Biochemical Technology Co. Ltd., China). The sections were baked in a chip dryer to increase tissue adhesion and deparaffinized and rehydrated using conventional methods. Endogenous peroxidase was blocked by treating the sections with 3% hydrogen peroxide (A0005077; Shanghai Runjie Chemical Reagent Co. Ltd., China) in methanol for several minutes. Tissue sections are placed in Citrate Antigen Retrieval Solution (C108873; Aladdin Reagent (Shanghai) Co. Ltd., China) and boiled continuously for 10 min to expose antigenic sites. Then the tissue sections were non‐specifically blocked with goat serum (A602440, Shengong Bioengineering Shanghai (Stock) Co. Ltd., China) for half an hour. Primary antibodies against MMP13 (1:100, SAB2104396; Sigma, USA), Runx2 (1:100, AV36678; Sigma), Ihh (1:50, SAB2108031; Sigma), ALP (1:200, ab95462; Abcam, USA), ANKH (1:50, PA5‐43526; Thermofisher, USA) were added dropwise and incubated at 4°C in the refrigerator overnight. After incubation with the primary antibody, the secondary antibody (A0208; Biyuntian Biotechnology Co. Ltd., China) is added dropwise and incubated for half an hour at 37°C. This was followed by colour development using the DAB kit (PA110; Tiangen Biochemical Technology Co. Ltd., China), and the sections were counterstained with haematoxylin. Photomicrographs were taken with a Nikon Ri 1 microscope (Nikon, Melville, NY, USA).

#### 2.2.4 Real‐Time RT‐PCR (RT‐qPCR)

The mRNA levels of IL‐1β, MMP13, Ihh, and Runx2 were quantified using RT‐qPCR. Total RNA from meniscus tissue in each group (*n* = 4) was isolated using Trizol reagent (15,596,018; Ambion, USA). Accordingly, 1 μg total RNA was reverse transcribed into cDNA using a High‐Capacity cDNA Reverse Transcription Kit (11123ES60; Shanghai Yisheng Biotechnology Co. Ltd., China), with reaction conditions: 25°C for 5 min, 42°C for 30 min, 85°C for 5 min, and kept at 4°C. Thereafter, 50 ng/μL of the resulting cDNA was used as the template to quantify the relative mRNA content using the Power SYBR Green PCR Master Mix (1120ES08; Shanghai Yisheng Biotechnology Co. Ltd.). qPCR was initiated for 5 min at 95°C, then 40 cycles of denaturation at 25°C for 10 s, primer annealing for 20 s at 42°C, and a final extension step at 85°C for 20 s. Primer pairs that were used for quantitative detection of gene expression are listed in Table 1, and β‐actin rRNA was used as the internal control. The cycle threshold values for target genes were measured and calculated using computer software (MJ Research; Bio‐Rad Laboratories Inc.). Relative transcript levels were calculated using the 2‐ΔΔCq method, where ΔΔCq = ΔCq E‐ΔCq C, ΔCq E = Cqexp‐Cq18S, and ΔCq C = CqCCq β‐actin.

**TABLE 1:** Primer sequences for reverse transcription‐polymerase chain reaction used in vivo experiment.

Gene	Primer sequence, 5′‐3′
Ihh	Forward: CATCTCCGTCATGAATCAGT Reverse: TCCAGGAAAATGAGCACGTC
IL‐1β	Forward: GGATCAAGCTGCAAATCTCC Reverse: TTGTCGGTTCAGATTGTCTCC
Runx2	Forward: CCAGAGCGGACCTTTCCA Reverse: GATCCCGACGAAGTGCCATA
MMP13	Forward: CAGTTGTACATGCCCCTCTTCA Reverse: TCCAAAGCCACATATACCATCCT
β‐Actin	Forward: GGCGCTTTTGACTCAGGATTTA Reverse: GATGCTTGCTCCAACCAACTG

### 2.3 In Vitro Rat Menisci Study

#### 2.3.1 Isolation, Culture, and Immunofluorescence Identification of PMFs

Medial and lateral menisci were isolated from one‐month‐old rat knees (*n* = 6) that were procured from Shanghai University of Chinese Medicine Laboratory Animal Centre. Specific methods refer to previous studies [33]. The phenotype of PMFs after 48 h in culture was identified. PMFs were fixed in 4% paraformaldehyde for 20 min after cell crawling. Then 0.5% Triton X‐100 (A110694; Sangon Biotech, China) was added dropwise to permeabilise the cells. The cells were blocked using 1% BSA (A600332; Sangon Biotech) at room temperature for 30 min and subsequently were added dropwise specific primary antibody (Collagen II: ab34712; Abcam; Collagen X: ab58632; Abcam) at 4°C in a wet box overnight. The following day, the cells were washed with PBS (G002; Servicebio, China) and incubated in tetramethylrhodamine (TRITC)‐conjugated secondary antibodies in darkness for 1 h. Finally, 4′,6‐diamidino‐2‐phenylindole (DAPI, 1:1000 dilution, 500 μg/mL, E607303; Sangon Biotech) was used for nuclear staining for 30 min. Immunofluorescent images were captured using an Olympus IX70 Inverted Microscope (Tokyo, Japan).

#### 2.3.2 Cell Proliferation Assay

Cell counting kit‐8 (CCK‐8) was used for the evaluation of cell proliferation. PMFs were seeded into a 96‐well plate at a density of 100 μL per well and incubated in a cell incubator for 8 h. PMFs were treated with varying concentrations of HBP‐A (0.01, 0.025, 0.05, 0.1, 0.2, 0.3, 0.4, 0.5, 0.6, and 1 mg/mL, respectively). Then, 100 μL CCK‐8 solution (C0038; Beyotime) was added to each well and maintained in a 37°C incubator for 1 h. Finally, the absorbance of each well was measured at 450 nm using an enzyme marker (INFINITE M NANO, TECAN, Germany).

#### 2.3.3 Mechanical Stimulation

The Flexcell FX‐5000 TensionSystem (FX5K; Flexcell International Corp., Hillsborough, NC, USA) was used to apply mechanical cyclic tensile stretch to PMFs [34]. The cell was subjected to continuous mechanical stimulation with a uniaxial sinusoidal waveform with 10% elongation and a frequency of 0.5 Hz for 30 min, 24 h, and 48 h, as reported previously [34, 35]. Each cycle consisted of 10s strain and 30s relaxation. Control cultures grown under the same conditions but without the strain protocol. Western blot and quantitative analysis were showed the expression of MMP13 at different points in time. Successful model identification was followed by a 24 h intervention using 0.3 mg/mL HBP‐A and p38‐MAPK signalling pathway inhibitor SB203580. CCK‐8 was used for the evaluation of cell proliferation. Then samples are collected for subsequent testing and analysis.

#### 2.3.4 Apoptosis and Morphological Changes of PMFs

Flow cytometry analysis was evaluated for the percentage of apoptotic cells by staining cells with Annexin V‐FITC (C1062S; Beyotime). Staining by rhodamine‐labelled ghost pen cyclic (40734ES75; Yeasen, China) peptide showed the morphology of PMFs in different groups. Alizarin red (G1038; Servicebio, China) and toluidine blue staining (G1032; Servicebio) demonstrated the mineralisation and hypertrophy of the PMFs in different groups.

#### 2.3.5 Western Blot

The protein levels related to hypertrophy and degeneration (including MMP13, Ihh, and IL‐1β), mineralisation (including ANKH, Runx2, and ALP), cartilage degeneration (HDAC4), and p38‐MAPK signalling pathway (p38 and Caspase3) were determined by western blot. Total protein was extracted by homogenisation in a complete Lysis‐M kit (P0013C; Beyotime) from rat PMFs and quantified by the BAC Protein Assay Kit (C503021; Sangon Biotech). Equal amounts of protein lysates were separated by SDS‐polyacrylamide gel electrophoresis (SDS‐PAGE) and transferred to a nitrocellulose membrane for immunoblot analysis and stained with specific primary antibodies. The following primary antibodies were used: MMP13 (SAB2104396; Sigma), Ihh (SAB2108031; Sigma), ANKH (PA5‐43526; Thermofisher, USA), ALP (ab95462; Abcam), HDAC4 (5392; CST, USA), p38 (8690; CST), Caspase3 (9662; CST). Alexa Fluor 594 secondary antibodies (33112ES60; Yeasen) were detected with the ECL chemiluminescence test kit (36208ES76; Yeasen).

#### 2.3.6 Real‐Time RT‐PCR (RT‐qPCR)

The mRNA levels associated with hypertrophy and degeneration, mineralisation and cartilage degeneration, and the p38‐MAPK signalling pathway were quantified by RT‐qPCR. The detection method is the same as in vivo experiments. qPCR was initiated for 5 min at 95°C, then 40 cycles of denaturation at 95°C for 10 s, primer annealing for 20 s at 55°C, and a final extension step at 72°C for 20 s. Primer pairs that were used for quantitative detection of gene expression are listed in Table 2, and GAPDH rRNA was used as the internal control.

**TABLE 2:** Primer sequences for reverse transcription‐polymerase chain reaction used in vitro experiment.

名称	引物序列 (5′‐3′)
p38	Forward: GGATATTTGGTCCGTGGGCT Reverse: CGCATTATCTGCTGAAGCTGG
Caspase‐3	Forward: GCTGGACTGCGGTATTGAGA Reverse: GCGTACAGTTTCAGCATGGC
HDAC4	Forward: ACCGCTATGACGATGGGAAC Reverse: ACCACATCTGGGGCAAACTC
MMP13	Forward: GAGATGAAGACCCCAACCCTAA Reverse: AGGGCTGGGTCACACTTCTCT
Ihh	Forward:AGACCGCGACCGAAATAAGT Reverse:CACACGCTCCCCAGTTTCTA
IL‐1β	Forward: GGCAGCATTGTCGACAGAAGA Reverse: GCACTGGTCCAAATTCAATTCA
Runx2	Forward: CTCTGACTTCTGCCTCTGGC Reverse: ACCACATCTGGGGCAAACTC
ANKH	Forward: TTGGAGTGGACTTCGCCTTT Reverse: TCTCCCACAAACCCTGCTAGA
ALP	Forward: AGGACACGCTAACGCTCATC Reverse: CTGCCTGCTGCTTGTAGTTG
GAPDH	Forward: TGGCCTCCAAGGAGTAAGAAAC Reverse: GGCCTCTCTCTTGCTCTCAGTATC

### 2.4 Statistical Analysis

All results were expressed as means ± standard deviation (mean ± SD). Statistical analysis was performed using Students t test, and *p* < 0.05 was considered statistically significant. Statistics were performed using SPSS 22.0 software.

## 3 Results

### 3.1 HBP‐A Reduced Excessive Hypertrophy and Pathological Mineralisation of the Menisci Caused by Abnormal Mechanical Damage After ACLT

**FIGURE 1:**
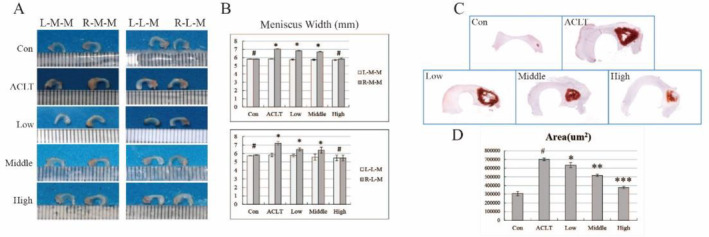
HBP‐A slows down meniscal hypertrophy and mineralisation. Right knee joints: ACLT group. The left joints: Self‐controlled study. Con: Normal guinea pig. (A, B) Width measurement and quantification analysis of hypertrophy of medial and lateral meniscus after intervention with different concentrations of HBP‐A. Quantitative comparison of the left and right knee joints in the medial or lateral menisci. # vs left knee, *p* > 0.05; * vs left knee, *p* < 0.05. L‐M‐M, Left Medial Meniscus; R‐M‐M, Right Medial Meniscus; L‐L‐M, Left Lateral Meniscus; R‐L‐M, Right Lateral Meniscus. (C, D) Alizarin red staining and quantification analysis showed meniscal mineralisation and its area of the right medial meniscus after intervention with different concentrations of HBP‐A. Data are shown as mean ± SD, *n* = 4, # vs Con, *p* < 0.05; *, **, *** vs ACLT, *p* < 0.05.

Our previous studies have concluded that the hypertrophy and mineralisation of the meniscal tissue are associated with cartilage degeneration caused by abnormal mechanical damage after ACLT [22]. Consistent with this, there was a significant increase in meniscal width, mineralised area, and intensity in the ACLT group compared to the control group (Figure 1). After intervention with different concentrations of HBP‐A, we found varying degrees of improvement in medial and lateral meniscal width of the right knee in all HBP‐A intervention groups compared to the ACLT group, and the meniscal width gradually improved with the increase of HBP‐A concentration. And the most significant improvement in meniscal width was observed in the high‐concentration group (Figure 1A,B). It is worth mentioning that the meniscal width between the high‐concentration and control groups was not significantly different (Figure 1A,B). Subsequently, we used Alizarin red staining to evaluate the right knee's mineralisation of the medial menisci (stress concentration areas). Alizarin red staining and quantification analysis (Figure 1C,D) showed a reduction in the area of mineralisation after HBP‐A intervention. Interestingly, these areas of mineralisation are concentrated on the medial aspect of the anterior horn of the medial meniscus.

### 3.2 HBP‐A Mitigated Damage Degeneration of the Right Medial Meniscus and Tibial Articular Cartilage Caused by Abnormal Mechanical Damage After ACLT

**FIGURE 2:**
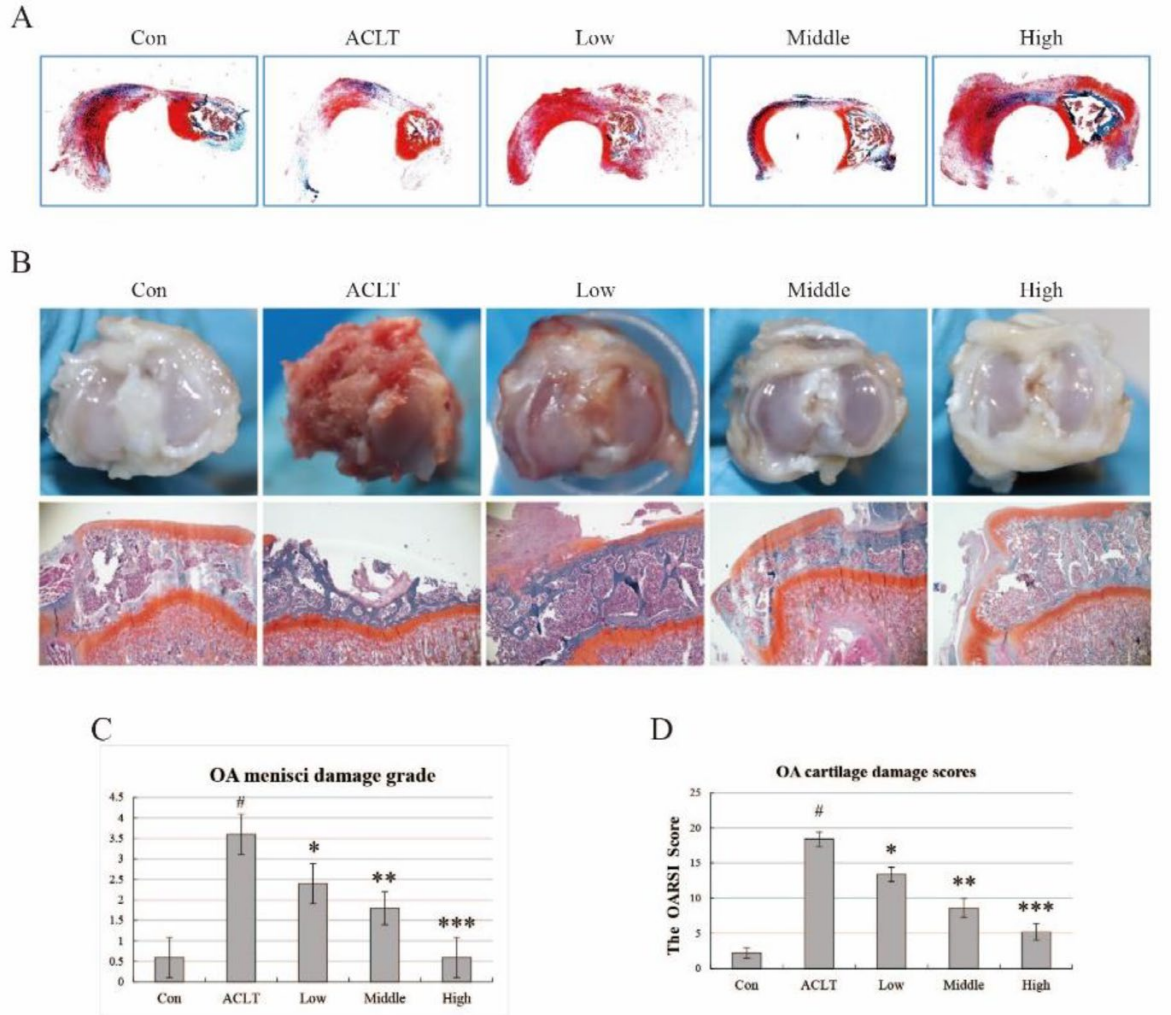
HBP‐A reduced the degeneration of the right medial meniscus and tibial articular cartilage. (A, C) Safranin O/Fast Green staining and OA menisci damage grade exhibited the damage of medial meniscus of the right knee joints and the effect of different concentrations of HBP‐A intervention. (B, D) The overall shape and Safranin O/Fast Green of the tibial articular cartilage and OA cartilage damage scores depicted the damage of the tibial articular cartilage of the right knee joints and the effect of different concentrations of HBP‐A intervention. Data are shown as mean ± SD, *n* = 4, # vs Con, *p* < 0.05; *, **, *** vs ACTL, *p* < 0.05.

We evaluated the degree of damage to the right medial meniscus and tibial articular cartilage using Safranin O/Fast Green staining. The staining showed the degree of damage to the right medial meniscus had significantly increased in the ACLT group compared with the control group. We found severe wear, tearing, and substantial loss of polysaccharides at the medial edge of the meniscus in the ACLT group, while only minor damage and mild loss of proteoglycans were found in the control group (Figure 2A). And the area of damage was mainly concentrated in the medial anterior horn of the medial meniscus (stress concentration areas), which was consistent with the results of the Alizarin red and Von Kossa staining. Damage in the HBP‐A treatment group was progressively improved in a concentration‐dependent manner (Figure 2A). The OA menisci damage grade showed that the meniscal damage grade in the ACLT group was increased compared to the control group, and the meniscal damage grade in all HBP‐A intervention groups decreased compared to ACLT (Figure 2C). Among the HBP‐A intervention groups, the damage grade in the group treated with a high concentration of HBP‐A was significantly reduced. Consistent with previous results, the degree of damage mitigation was proportional to HBP‐A concentration.

Meanwhile, each group's overall shape of the tibia articular cartilage is shown below (Figure 2B). The Safranin O/Fast Green staining of tibial articular cartilage showed structural tearing and loss of proteoglycans in the surface, middle, and deep layers of the tibial articular cartilage, with cell aggregation and hypertrophy. Necrosis in the ACLT group compared to the control group, only minor damage and mild loss of proteoglycan were found in the tibial articular cartilage (Figure 2B). The tibial articular cartilage damage and proteoglycan loss gradually improved in treatment groups with different concentrations of HBP‐A, showing consistency in concentration (Figure 2B). OA cartilage damage scores showed an increase in the tibial articular cartilage in the ACLT group compared to the control group, and the cartilage damage score of the tibial articular cartilage in the HBP‐A intervention group was reduced compared to ACLT (Figure 2D). And the damage score decreased significantly with increasing HBP‐A concentrations, which is consistent with previous results of meniscal damage grade.

### 3.3 HBP‐A Reduced the Overexpression of Protein and mRNA Markers Associated With Pathological Hypertrophy and Mineralisation Caused by Abnormal Mechanical Damage After ACLT

**FIGURE 3:**
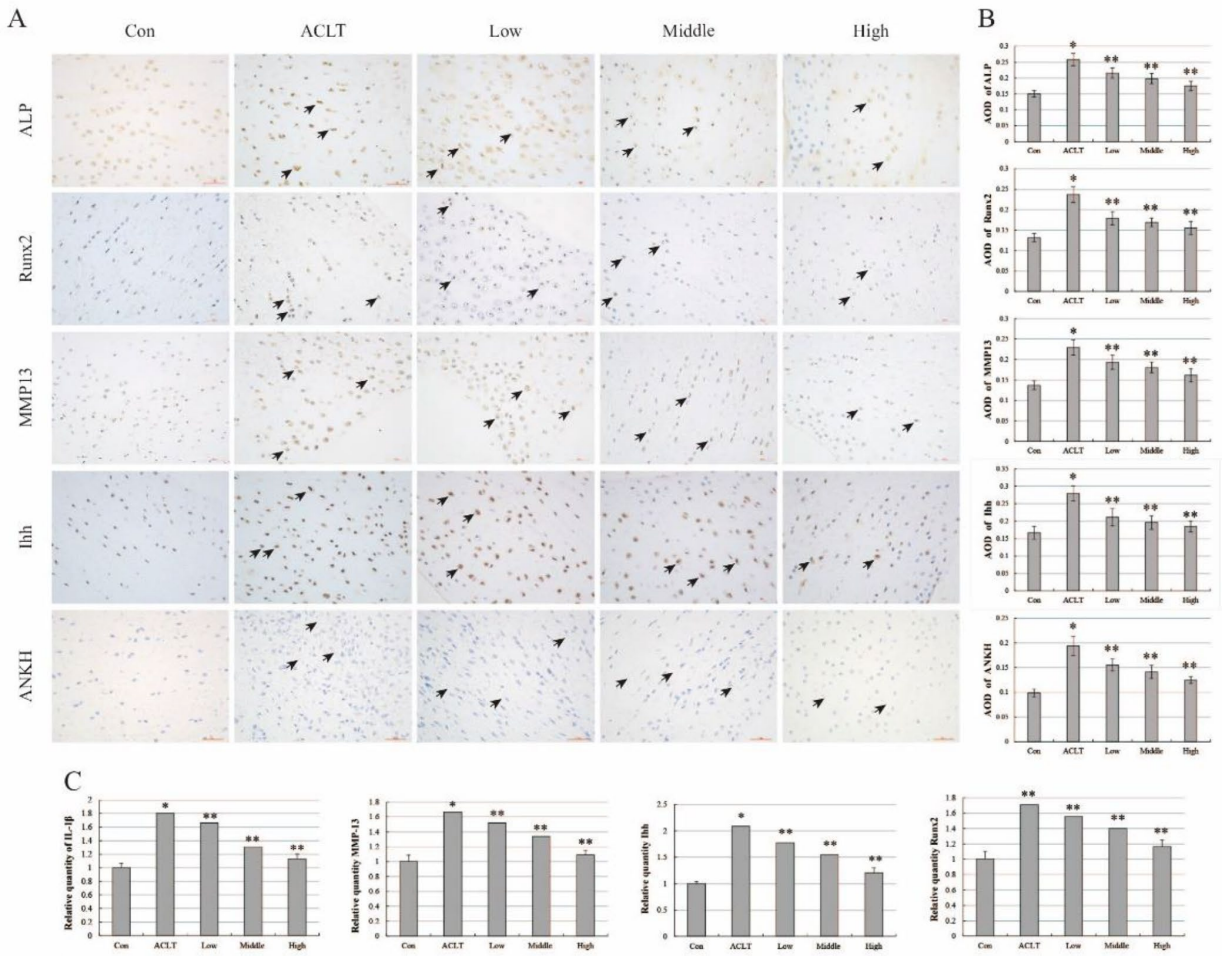
HBP‐A reduced overexpression of relevant markers related to pathological hypertrophy and mineralisation. (A, B) Immunohistochemical staining and its quantitative analysis showed expression situations of hypertrophy‐related protein markers MMP13, Ihh, and Mineralisation‐related markers Runx2, ALP, ANKH in the medial meniscus. Positive signals: Claybank particles (show by black arrow). (C) RT‐qPCR showed the mRNA expression levels of meniscus hypertrophy marker IL‐1β, MMP13, Ihh, and mineralisation marker Runx2. * vs Con, *p* < 0.05; ** vs ACTL, *p* < 0.05.

To confirm the morphological changes, we detected the expression of hypertrophy and mineralisation protein markers in the right meniscal tissue with or without HBP‐A interventions. Immunohistochemical staining and its quantitative analysis showed the number of positive hypertrophy protein markers MMP13, Ihh, and mineralisation protein markers ALP, Runx2, and ANKH was obviously increased in the ACLT group compared to the control group and decreased in the HBP‐A intervention group compared to the ACLT group. After intra‐articular injection of different concentrations of HBP‐A, the number of positive MMP13, Runx2, Ihh, ALP, and ANKH proteins expressed in each group gradually decreased in a concentration‐dependent manner (Figure 3A,B). In parallel, we also verified the expression of markers of meniscal hypertrophy and mineralisation at the mRNA level. As expected, RT‐qPCR results showed that the mRNA level of hypertrophy markers IL‐1β, MMP13, Ihh, and mineralisation markers Runx2 exhibited increased expression in the ACLT group compared to the control group, and reduced expression in the HBP‐A intervention group compared to the ACLT group. After different concentrations of HBP‐A intervention, the expression of mRNA levels of IL‐1β, MMP13, Ihh, and Runx2 in each group also gradually decreased and was consistent with the concentration of HBP‐A (Figure 3C). As expected, the expression trend of the mRNA level is consistent with the protein level.

### 3.4 0.3 mg/mL HBP‐A Improved the Inhibition of PMF Proliferation Induced by 10% Stretch

**FIGURE 4:**
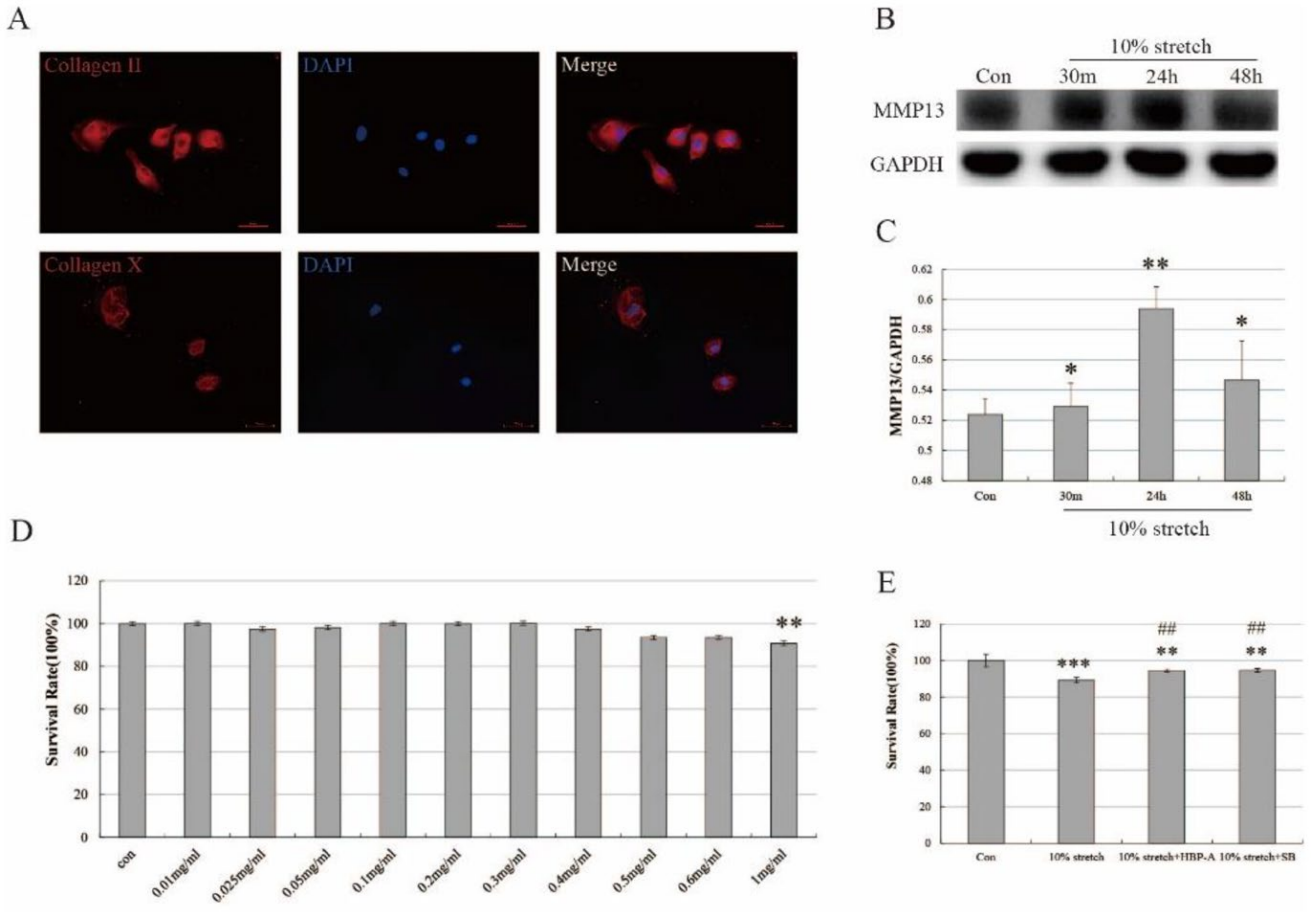
0.3 mg/mL HBP‐A improved inhibition of PMF proliferation in vitro. (A) Immunofluorescence showed high expression of collagen type II and V in all cultured PMFs (red fluorescent: collagen II or X in the cytoplasm; blue fluorescent: nucleus). (B, C) Western blot and quantitative analysis showed expression of MMP13 in PMFs at different time points after 10% stretch intervention. ** vs Con, *p* < 0.05; * VS Con, *p* > 0.05. (D) CCK‐8 assays showed the effect of HBP‐A on the proliferation of PMFs. ** vs Con, *p* < 0.05. (E) CCK‐8 assay showed 0.3 mg/mL HBP‐A and SB203580 improved the inhibition of 10% stretch‐induced proliferation of PMFs. ** vs Con, *p* < 0.05; *** vs Con, *p* < 0.05; ## vs 10% stretch, *p* < 0.05.

Our previous experiments constructed a method for the isolation and culture of rat meniscal fibrous chondrocytes in vitro and validated their biological properties [36]. After 48 h incubation of isolated rat PMFs, the cultured cells in vitro were identified by immunofluorescence to detect the chondrocyte‐specific markers, Collagen II and Collagen X. Immunofluorescence showed high expression of collagen type II and X in all cultured PMFs, consistent with the biological characteristics of fibrochondrocytes (Figure 4A).

We detected the expression of MMP13, an essential indicator of cartilage degeneration, in rat PMFs after 10% stretching force at 30 min, 24 h, and 48 h of intervention to determine the best way to model. Western blot and quantitative analysis showed the expression of MMP13 was significantly promoted after 24 h of 10% stretch intervention compared to the control group. In comparison, MMP13 expression had some change but no statistical difference after 48 h and 30 min of intervention (Figure 4B,C). Therefore, subsequent experiments used a 10% stretch intervention for 24 h as a model for PMF degeneration.

At the same time, we investigated the effect of different concentrations of HBP‐A on PMFS proliferation. CCK‐8 showed inhibition of the cell proliferation starting at 0.4 mg/mL HBP‐A when PMFs were treated with varying concentrations of HBP‐A (0.01, 0.025, 0.05, 0.1, 0.2, 0.3, 0.4, 0.5, 0.6, and 1 mg/mL, respectively) (Figure 4D). Combined with the previous research of our subject group [37], a concentration of 0.3 mg/mL of HBP‐A was identified for use subsequent experiments. Whereafter, PMFS induced by 10% stretch was intervened with 0.3 mg/mL HPB‐A, CCK‐8 showed cell proliferation in the 10% stretch group was significantly reduced compared to the control group. In contrast, cell proliferation in the HBP‐A intervention and pathway inhibitor groups was partially enhanced (Figure 4E).

### 3.5 0.3 mg/mL HBP‐A Improved Apoptosis and Morphological Changes of PMFS Induced by 10% Stretch

**FIGURE 5:**
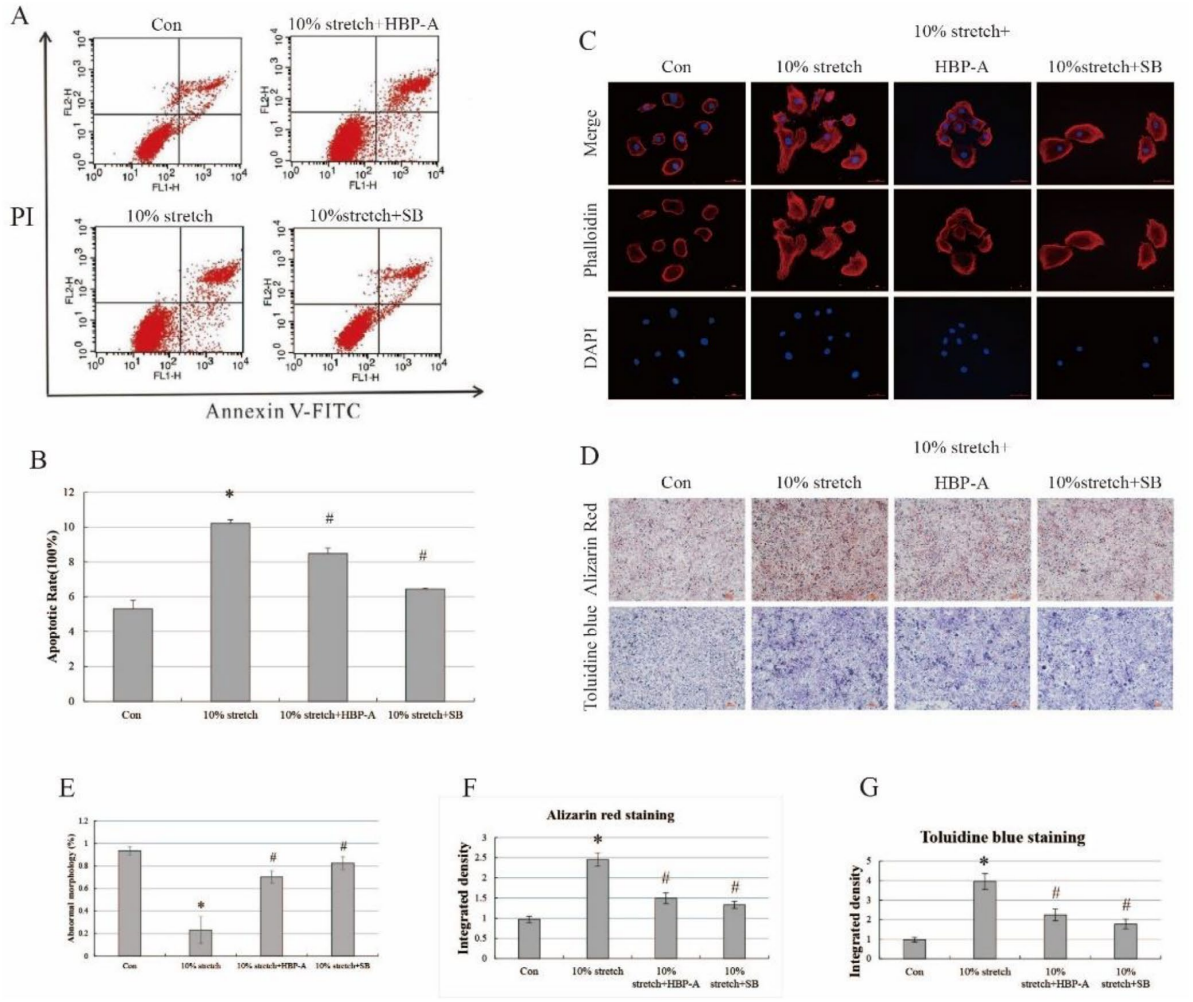
0.3 mg/mL HBP‐A improved apoptosis and morphological changes of PMFs in vitro. (A, B) Flow cytometry and analysis showed a significant increase in PMF apoptosis in the 10% stretch group and that 0.3 mg/mL HBP‐A and SB differentially reduced PMF apoptosis induced by 10% stretch. (C, E) Staining by rhodamine‐labelled ghost pen cyclic peptide showed the morphology of PMFs in different groups. (D, F, G) Alizarin red and toluidine blue staining demonstrated the mineralisation and hypertrophy of the PMFs in different groups. * vs Con, *p* < 0.05, # vs 10% stretch.

We investigated the effect of HBP‐A intervention on apoptosis and morphological changes in PMFs induced by a 10% stretch. Flow cytometry found that PMF apoptosis was significantly increased in the 10% stretch group compared to the control group and was obviously reduced in the 0.3 mg/mL HBP‐A intervention and SB203580 group compared to the 10% stretch group (Figure 5A,B). Staining of filamentous actin (F‐actin) by rhodamine‐labelled ghost pen cyclic peptide was used to observe the morphological changes of the PMFs. The results showed that the morphology of PMFs is full, rounded, and homogeneous in the control group, a 10% stretch intervention altered the morphology of the cells to a long spindle shape, and 0.3 mg/mL HPB‐A and SB203580 partially reversed the morphological changes induced by the 10% stretching force (Figure 5C,E). The mineralisation and hypertrophy of the PMFs were evaluated using Alizarin red and toluidine blue staining. Interestingly, two stainings showed the same trend, and results showed that the mineralisation and hypertrophy of PMFs in the 10% stretch group were significantly increased. At the same time, HBP‐A and SB203580 alleviated the mineralisation and hypertrophy of PMFs induced by 10% stretch (Figure 5D,F,G).

### 3.6 HBP‐A Altered Effectively the Expression of Genes Related to Hypertrophy and Mineralisation in PMFs by Suppressing the Overexpression of the p38‐MAPK Signalling Pathway

**FIGURE 6:**
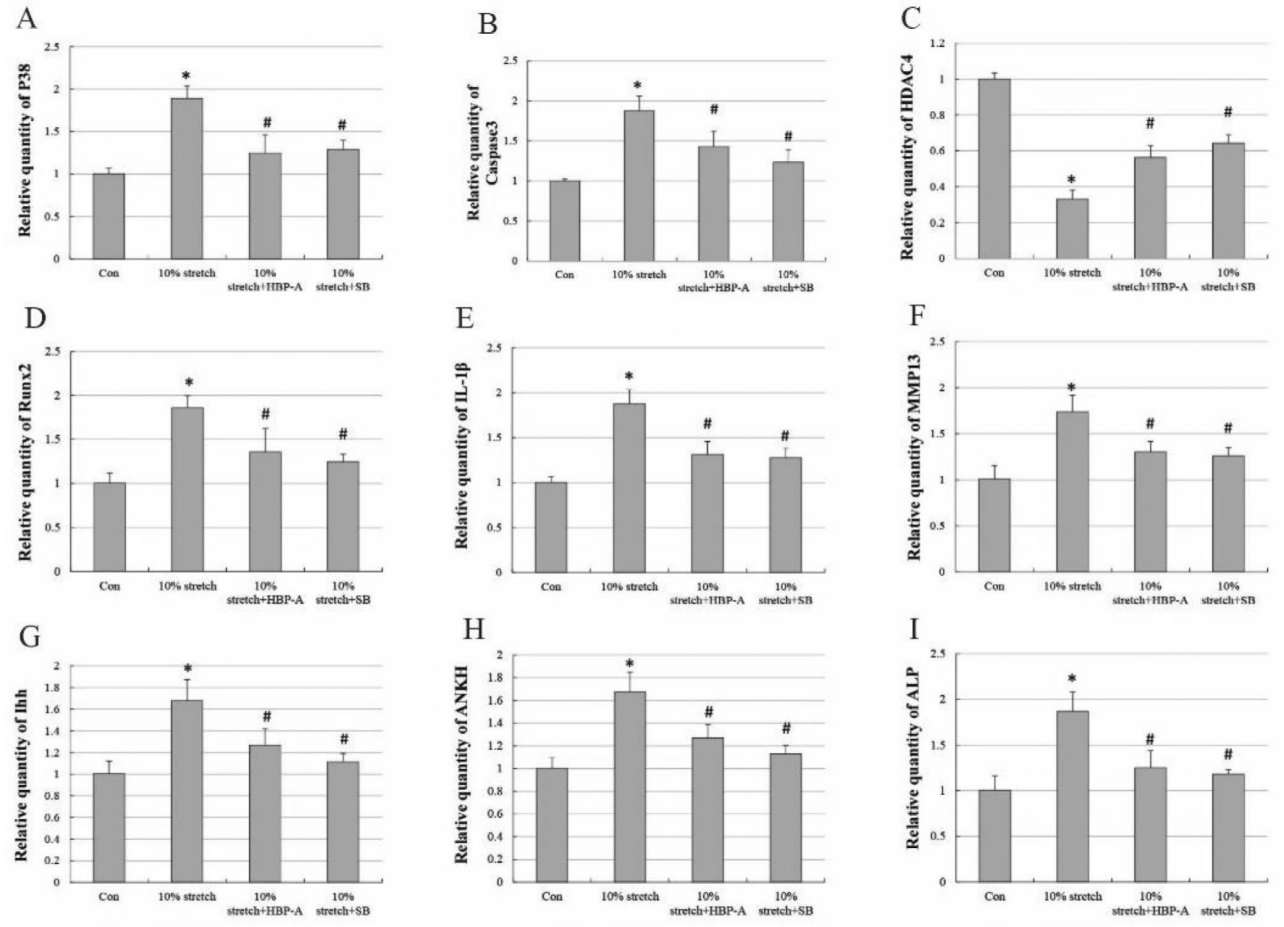
HBP‐A also altered the expression of genes associated with hypertrophy, mineralisation, and the P38‐MAPK signalling pathway in PMFs. (A–I) RT‐qPCR showed that the overexpression of the genes associated with excessive hypertrophy, degeneration, and mineralisation of PMFs resulted from 10% stretch force in vitro and that HBP‐A reduces hypertrophy and mineralisation of PMFs by inhibiting the p38‐MAPK signalling pathway. * vs Con, *p* < 0.05, # vs 10% stretch, *p* < 0.05.

To investigate the mechanism of HBP‐A in preventing hypertrophy and mineralisation of PMFs caused by abnormal mechanical damage, we detected the expression level of genes related to hypertrophy and calcification and p38‐MAPK signalling pathway‐related target markers using RT‐qPCR. The results showed that the expression levels of the genes related to excessive hypertrophy and denaturation (MMP13, Ihh) and mineralisation (ALP and ANKH) have a significant increase and that the expression levels of genes related to cartilage degeneration (HDAC4) have obviously decreased in the 10% stretch group compared to the control group. Noteworthy, the expression level of p38, a key target of the p38‐MAPK signalling pathway, and Caspase3, the downstream substrate of this signalling pathway, also has a significant increase in the 10% stretch group compared to the control group. After intervention with HPB‐A and the pathway inhibitor SB203580, the expression of MMP13, Ihh, ALP, ANKH, p38, and Caspase3 was significantly lower, and HDAC4 markedly higher than in the 10% stretch force group, with statistically significant differences. These results suggest that HBP‐A may have down‐regulated the overexpression of the p38‐MAPK signalling pathway to reduce the pathological degeneration of PMFs (Figure 6A–I).

### 3.7 HPB‐A Altered the Expression Level of Protein Associated With Mineralisation and Hypertrophy and p38‐MAPK Signalling Pathway in PMFs

**FIGURE 7:**
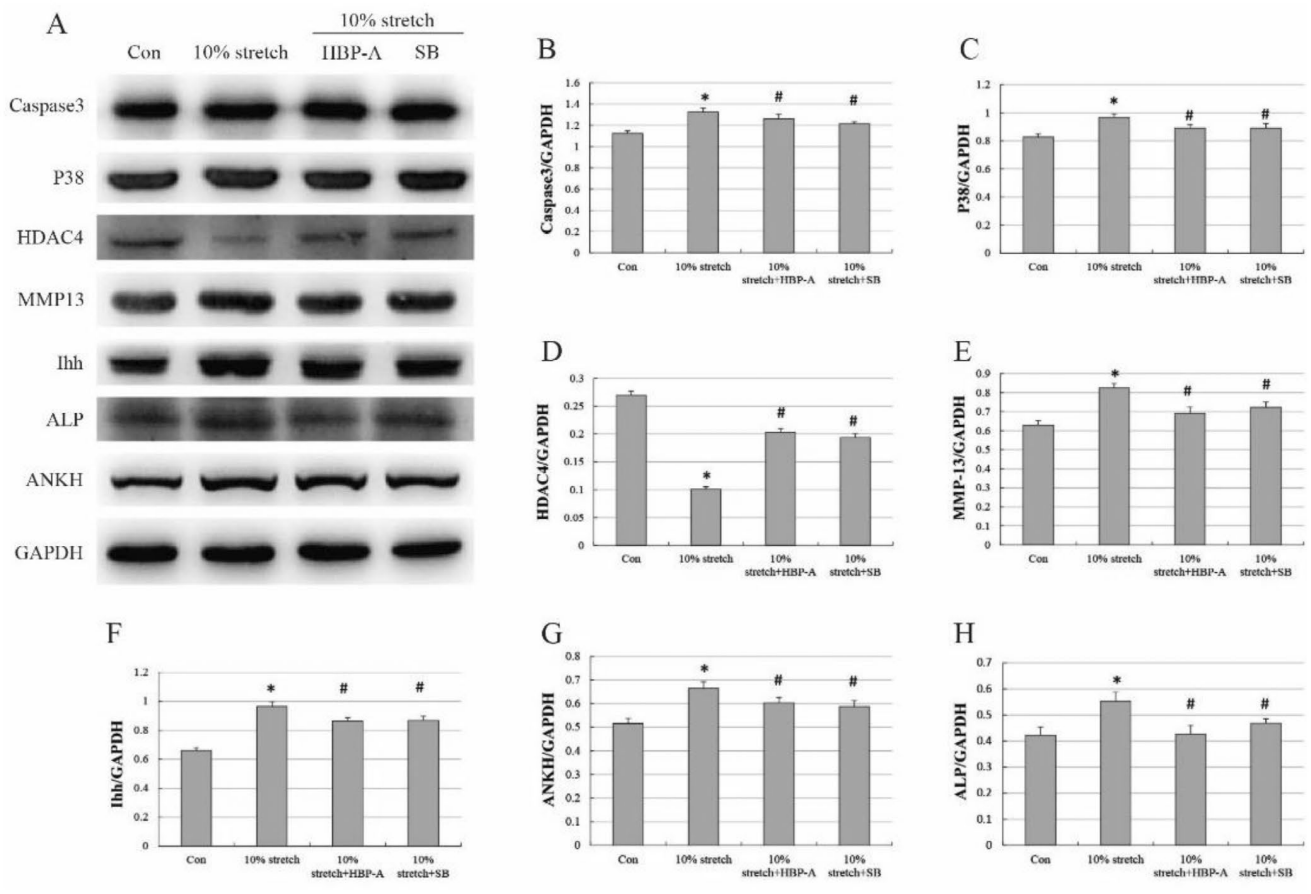
HBP‐A altered the protein expression related to hypertrophy, mineralisation, and the P38‐MAPK signalling pathway in PMFs. (A–H) Western and analysis showed that the overexpression of the protein markers associated with excessive hypertrophy, degeneration, and mineralisation of PMFs resulted from 10% stretch force in vitro and that HBP‐A and inhibitors reduced hypertrophy and mineralisation of PMFs by inhibiting the p38‐MAPK signalling pathway. * vs Con, *p* < 0.05, # vs 10% stretch, *p* < 0.05.

Finally, we detected the expression level of protein associated with hypertrophy and mineralisation of PMFs induced by abnormal mechanical damage by Western blot. As expected, the expression of the protein associated with hypertrophy and calcification is upregulated in the 10% stretch group. After treatment with HBP‐A and SB, the protein expression associated with hypertrophy and mineralisation and the p38‐MAPK signalling pathway was significantly lower, and cartilage degeneration‐associated protein HDAC4 was substantially higher compared to the 10% stretch group, which was consistent with the trend in gene level expression (Figure 7).

## 4 Discussion

For the treatment of KOA, Chinese medicine plays an increasingly integral role. Huaizhen Yanggan Capsule is an experienced formula for the dialectical therapy of KOA based on Chinese medicine theory by Professor Shi Yinyu, with remarkable clinical efficacy [38, 39]. HBP‐A, as the main active ingredient of mussel meat, which is the main component of this Chinese medicine, can inhibit the expression of MMP13 in chondrocytes and prevent cartilage degeneration [37]. However, the exact molecular mechanism of HBP‐A for treating KOA is still unclear. The main objective of this study is to investigate the molecular mechanism of HBP‐A for the treatment of KOA.

Our previous studies have demonstrated that abnormal mechanical stimuli lead to the upregulation of meniscal degeneration markers, thereby exacerbating the pathological process of knee osteoarthritis [22]. In this study, our results demonstrate that HBP‐A down‐regulates the expression levels of MMP13, Runx2, Ihh, ALP, and ANKH, which are specific markers for hypertrophy and mineralisation. Pathological staining showed significant improvement in meniscal hypertrophy and calcification after HBP‐A intervention. RT‐qPCR and immunohistochemistry verified these results. The results of in vivo experiments were further validated by in vitro experiments, where HBP‐A was shown to significantly alleviate additional stretch‐induced mineralisation and hypertrophy of meniscal fibrochondrocytes. These results suggested HBP‐A has a negative regulatory effect on pathological changes in the meniscus. Previous studies have indicated that HBP‐A has a therapeutic effect on osteoarthritis of the rabbit knee and promotes chondrocyte proliferation; the mechanism may promote chondrocyte type II collagen synthesis and delay chondrocyte degeneration by reducing the expression of the Wnt/β‐catenin signalling pathway [29, 37]. Similar results were found in our study, OA cartilage damage scores showed a significant reduction in cartilage damage and pathological degeneration after HBP‐A intervention. In addition, we also found an interesting phenomenon that meniscal calcification in the guinea pig with KOA is mainly concentrated in the anterior horn of the medial meniscus, which is consistent with the main location of meniscal calcification in KOA patients as previously reported [40].

In the pathogenesis of OA, the hypertrophy and mineralisation of the meniscus can lead to changes in cartilage production, which can affect joint stability and load transfer, thus accelerating the progression of OA. Pathological changes in the meniscus are also a significant cause of limited knee motion and pain [41]. Related studies have shown that calcification of the meniscus leads to the narrowing of the joint space and friction with the cartilage, which is a critical pathological factor in the further development and progression of KOA [40]. In this study, our results of Safranin O/Fast Green staining showed a consistent increase between OA menisci damage grade and OA cartilage damage scores after abnormal mechanical loading, both of which were significantly reduced after HBP‐A intervention. This suggests that meniscal damage is closely related to cartilage and may precede cartilage degeneration, consistent with previous findings [4, 42, 43, 44]. However, the specific relationship of injury mechanisms between the meniscus and cartilage requires more in‐depth studies. Previous studies have demonstrated the protective effect of HBP‐A on cartilage degeneration [29, 37]. In this study, we have further investigated the impact of HBP‐A on delaying meniscal hypertrophy and calcification.

Various studies have shown that the p38 MAPK signalling pathway is important in mechanical signal transduction [45, 46, 47, 48]. The p38 MAPK, as a vital component of the MAPK family, regulates cell differentiation, proliferation, cytokine production, senescence, and apoptosis and plays important roles in bone tissue homeostasis and development [49, 50, 51, 52]. Various factors, such as osmotic stress, cytokines, death receptors, UV, and oxidative stress, have been reported to activate this signalling pathway, with osmotic stress playing a significant role [53, 54]. One study found that cyclic compression of isolated meniscal explants in vitro activates the p38 signalling pathway [23]. Our study found that abnormal mechanical loading led to overexpression of p38 and caspase‐3 and promoted down‐regulated HDAC4. These data verified that abnormal mechanical injury could activate the p38 MAPK signalling pathway, leading to overexpression of markers associated with meniscal hypertrophy and calcification. These results demonstrate that abnormal mechanical damage can activate the p38 MAPK signalling pathway leading to overexpression of the markers related to hypertrophy and calcification, thus allowing for pathological changes in the meniscus. Interestingly, overexpression of target proteins related to the p38 MAPK signalling pathway associated with the meniscus was significantly suppressed after HBP‐A treatment. These results confirm our previous speculation. It has been reported that p38‐MAPK induces the degradation of histone deacetylase HDAC4 by upregulating the expression of its downstream substrate apoptosis protein‐3 (caspase‐3), which in turn increases the expression of Runx2 and MMP13 markers associated with cartilage degeneration [26]. The role of HDAC4 in cartilage protection and prevention of cartilage degeneration is a continuing focus of research into the mechanisms of KOA [55, 56, 57]. However, its mechanism of meniscal degeneration and protection is poorly studied. This study showed that HDAC4 levels were significantly reduced in PMFs induced by abnormal mechanical stretch, which is consistent with the previously reported trend of HDAC4 in cartilage in the literature [58]. At the same time, we found that HDAC4 levels in PMFs were significantly increased after 0.3 mg/mL HBP‐A intervention. These results suggest that the mechanism by which HBP‐A slows down meniscal hypertrophy and mineralisation may be achieved by inhibiting the excessive activation of the p38 MAPK signalling pathway and preventing the degradation of HDAC4.

There are several limitations to our research study. One limitation is that mechanistic validation of in vitro experiments was not performed. It is well known that in vitro experiments further validate in vivo experiments. In this experiment, the phenotype of HBP‐A action was investigated in vitro in guinea pigs and further validated in vitro in rat PMFs. Still, unfortunately, no mechanistic validation was performed in the in vivo experiments. Further in‐depth studies are needed. A further limitation is that the mechanism of action of HBP‐A needs to be further investigated. In this experiment, we verified that the mechanism by which HBP‐A delays meniscal hypertrophy and calcification occurs through inhibition of excessive activation of the p38 MAPK signalling pathway, but how HBP‐A activates the p38 signalling pathway requires further more in‐depth study.

## 5 Conclusion

This study verified the specific signal transduction mechanism of meniscal hypertrophy and calcification that abnormal mechanical stimulation activates the p38‐MAPK signalling pathway and also explored the molecular mechanism of the protective effect of HBP‐A on the pathological degeneration of the meniscus by regulating the p38‐MAPK signalling pathway. Meniscal damage is an early event in the development of KOA pathology. This study reveals the potential therapeutic role of HBP‐A on KOA based on meniscal hypertrophy and calcification.

## Author Contributions

**Zhibi Shen:** conceptualization (equal). **Zongrui Yang:** writing – original draft (equal); formal analysis (equal); methodology (equal); data curation (equal); validation (equal). **Yuanyuan Feng:** writing – original draft (equal); investigation (equal); visualization (equal). **Mingcai Zhang:** writing – review and editing (equal); funding acquisition (equal). **Yongming Liu:** methodology (equal). **Yizhe Xiong:** funding acquisition (equal). **Xiang Wang:** investigation (equal). **Ying Shi:** visualization (equal). **Bo Chen:** investigation (equal). **Zhengming Wang:** formal analysis, data curation (equal). **Haiya Ge:** formal analysis, methodology (equal). **Hongsheng Zhan:** resources (lead); funding acquisition (lead); conceptualization (lead); project administration (lead). **Zhibi Shen:** writing – review and editing (lead); project administration (lead); validation (lead); supervision (lead). **Guoqing Du:** resources (lead); writing – review and editing (lead); funding acquisition (lead); conceptualization (lead); validation (lead).

## Conflicts of Interest

The authors declare no conflicts of interest.

## Data Availability

The original contributions presented in the study are included in the article; further enquiries can be directed to the corresponding author(s).
